# Visualization and Measurements of Blood Cells Flowing in Microfluidic Systems and Blood Rheology: A Personalized Medicine Perspective

**DOI:** 10.3390/jpm10040249

**Published:** 2020-11-26

**Authors:** Diana Pinho, Violeta Carvalho, Inês M. Gonçalves, Senhorinha Teixeira, Rui Lima

**Affiliations:** 1 Center for MicroElectromechanical Systems (CMEMS-UMinho), Campus de Azurém, University of Minho, 4800-058 Guimarães, Portugal; 2 Research Centre in Digitalization and Intelligent Robotics (CeDRI), Campus de Santa Apolónia, Instituto Politécnico de Bragança, 5300-253 Bragança, Portugal; 3 MEtRICs, Mechanical Engineering Department, Campus de Azurém, University of Minho, 4800-058 Guimarães, Portugal; violeta.carvalho@dem.uminho.pt (V.C.); rl@dem.uminho.pt (R.L.); 4 Instituto Superior Técnico, Universidade de Lisboa, Av. Rovisco Pais, 1049-001 Lisboa, Portugal; id9385@alunos.uminho.pt; 5 ALGORITMI Center (CAlg), University of Minho, 4800-058 Guimarães, Portugal; st@dps.uminho.pt; 6 CEFT, Faculdade de Engenharia da Universidade do Porto (FEUP), Rua Dr. Roberto Frias, 4200-465 Porto, Portugal

**Keywords:** hemorheology, blood diseases, microfluidics, single-cell analysis, red blood cells deformability, blood analogues

## Abstract

Hemorheological alterations in the majority of metabolic diseases are always connected with blood rheology disturbances, such as the increase of blood and plasma viscosity, cell aggregation enhancement, and reduction of the red blood cells (RBCs) deformability. Thus, the visualizations and measurements of blood cells deformability flowing in microfluidic devices (point-of-care devices) can provide vital information to diagnose early symptoms of blood diseases and consequently to be used as a fast clinical tool for early detection of biomarkers. For instance, RBCs rigidity has been correlated with myocardial infarction, diabetes mellitus, hypertension, among other blood diseases. In order to better understand the blood cells behavior in microfluidic devices, rheological properties analysis is gaining interest by the biomedical committee, since it is strongly dependent on the interactions and mechanical cells proprieties. In addition, the development of blood analogue fluids capable of reproducing the rheological properties of blood and mimic the RBCs behavior at in vitro conditions is crucial for the design, performance and optimization of the microfluidic devices frequently used for personalized medicine. By combining the unique features of the hemorheology and microfluidic technology for single-cell analysis, valuable advances in personalized medicine for new treatments and diagnosis approach can be achieved.

## 1. Introduction

Blood viscosity and red blood cells (RBCs) deformability are the main contributors to the maintenance and regulation of microcirculation. Many studies have demonstrated that modifications in the hemorheological properties were closely related to several metabolic diseases. Hemorheology, the study of deformation and blood flow, have been more focused on RBC rheology, relating the deformation and aggregation of RBCs, since erythrocytes comprise the major components in blood. However, RBCs studies alone may not show the progress of the disease, thus complementary studies are needed, such as whole blood and plasma viscosity, RBC aggregation, deformability measurements, blood cells population count, and morphological analysis [1,2,3,4,5,6,7] must be integrated as to further develop an efficient point-of-care tool.

Blood and plasma viscosity are risk factors essentially, for example for atherosclerosis [1,8,9] and other studies exposed that RBCs rheological changes have been observed in patients with hypertension [1,8,10] and diabetes mellitus [11,12,13,14], which are diseases more often associated with obesity [8,15]. Obesity-related blood rheological disturbances are currently being investigated as one of the risk factors for several co-morbid pathologies because they play a significant role in microcirculation blood flow [8]. Lee et al., 2019 [6] have performed a review with the most recent clinical studies of diabetic kidney disease associated with hemorheological parameters, demonstrating that critical shear-rate and –stress, measured by a microfluidic aggregometry [6], aggregation index and RBC deformability elongation index, measured by a microfluidic ektacytometry [16], must be combined as a tool for a successful diagnosis of disease stage and possible derivate complications. Additionally, Caprari et al., (2019) [7] have demonstrated that blood viscosity and RBC aggregation increase with the decreasing of the RBCs deformability, by using blood samples from subjects with sickle cell anemia. However, no microfluidic technologies have been used in these studies. Likewise, the electrical blood behavior can be used to help in the isolation of plasma and populations of blood cells and to quantify hemorheological properties [17,18]. Note that the electrical properties of blood tend to change due to hemorheological variations of the RBCs behavior and blood plasma. Several impedance measurements can be related to RBCs behavior, for example, the electrical impedance of blood flow increases at low shear rates because of RBC aggregation. A clear example that the electrical behavior of blood can help in the clinical diagnosis is the work developed by Yeom et al., (2015) [17]. In this work, a simple speckle analysis based on a microfluidic measurement method to detect the hyperaggregation caused by diabetes was used. By this method they have demonstrated the potential of evaluating the differences of the biophysical properties of cardiovascular diseases’ blood samples [19]. However, the measurements were restricted on superficial vessels, and ultrasonic signal requires calibration as a function of the flow speed under steady flow conditions.

**Figure 1:**
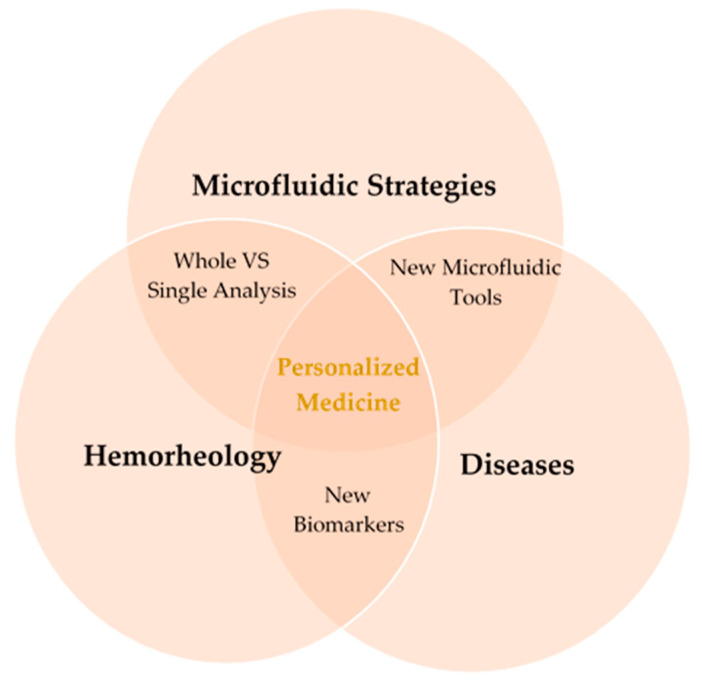
Hemorheology combined with microfluidic technology can bring further advances in personalized medicine for new treatments and diagnosis.

Therefore, the progress on hemorheology, regarding the phenomena associated with blood components, their interactions and impact on blood properties, and blood flow, i.e., the hemorheological profile of the patients, strongly dependent on the interactions and mechanical properties of blood cells and in particular with the behavior of the RBCs, can bring further insight into the human health state and it can be an important parameter in clinical diagnosis (Figure 1). Implementation of rapid test technologies in clinical environments leads to minimal user intervention during operation; user-friendly, easy-to-use, and simple detection platforms; high diagnostic sensitivity and specificity; immediate clinical assessment; and low manufacturing and consumables costs. However, it should be noted that it essential to achieve high specificity in the detection and read of biomarkers. Microfluidics has potential to provide all that solutions since it enables the processing of samples that are not available in large quantities (e.g., cells from patient biopsies), reduce cost, provides a high level of automation, and allows the set-up of complex models (for example for cancer studies). Another advantage more closely related to the rapid tests is the possibility of using small amounts of different kind’s body fluids such as blood, urine, saliva, and sweat.

Separation and sorting of different populations or subpopulations of blood cells from unprocessed or minimally processed blood specimens is of interest to both clinical and biomedical applications and holds a central role in diagnosis and prognosis of physiologic and pathologic conditions. In particular, plasma separation or cells separation from plasma has been achieved through several mechanisms, including acoustophoresis [20,21], dielectrophoresis [20,22], electrohydrodynamics [20,23], and physical hydrodynamic manipulation [20,24]. Several works have demonstrated that, by using hydrodynamic [25], hemodynamic [26,27,28], and physical filtration [25,29], high isolation efficiency is achieved. By physical filtration, more than 95% of the RBCs and 27% of the white blood cells (WBCs) are removed from whole blood [30], and this method is the most used to isolate and separated RBCs and WBCs from plasma. For the hydrodynamic manipulation several authors developed microfluidic devices that takes advantage of the intrinsic features of blood flow in the microcirculation, such as plasma skimming and leukocyte margination, to separate WBCs directly from whole blood. For example, Fujiwara et al., (2009) [31] and other authors have found evidence that it is possible to create an artificial cell-free layer (CFL) under appropriate hemodynamic and geometrical conditions, and also that CFL thickness is strongly influenced by the RBC deformability, which can be useful to separate plasma from the blood cells, as Pinho et al., (2013) [26] have also demonstrated.

However, pre- and post-sample preparation are still needed, and hemorheological properties most of the times are not quantified. One of the long term, goals in the field of microfluidics is to create integrated, portable clinical diagnostic devices for home and bedside use, eliminating time-consuming laboratory analysis procedures. Thus, it is imperative the development of integrated microfluidic devices, as described and implemented by Peng et al., 2019 [32], that combine several well-known methodologies from blood sample processing to cells population separation/sorting to single-cell hemorheological analysis, in order to achieve a powerful tool able to perform successful and accurate results. This opportunity to develop such complete tool will address new point-of-care systems and it can be a powerful not only for blood flow studies but also, for liquid biopsy handling and analysis.

## 2. Microfluidics Tools: Single-Cell Approach

Owing to recent developments in microfluidic technology, several hemorheological point-of-care devices have been designed that allow the possibility of conducting extensive clinical studies using hemorheological measurements [6,33]. The field of microfluidics technology is characterized by the study and manipulation of fluids in microstructures at the submillimeter length scale and it has emerged as an important tool to be applied in many engineering and biomedical fields [34]. The dimensions of such technology, normally less than 1 mm, conduct to useful methodologies with significant advantages, such as the volume of fluids within these channels (µl or even pL of sample) as well as, the reduction of the amount of necessary expensive reagents and analytes, sample loss, and dilution, but keeping their efficiency and high sensibility. The high-throughput for larger samples manipulation and analysis combined with parallel and multiple functionalities will avoid contaminations and handling errors. Additional advantages appear with the development of fabrication techniques used to design the microfluidic systems at the micro and nano dimensions. These techniques are relatively diversified and include inexpensive strategies, in particular with the progress of the 3D printing technology [35,36]. The cost reduction and the performance enhancement were appellatives for the biomedical research community to create novel strategies for applications in the diagnostics and/or therapy of several diseases, providing new sets of solutions to overcome the shortcomings of conventional detection and treatment methods available in clinics and hospitals [37].

The first routine in clinical practice is the complete blood cells count, determining the frequency of all major blood cells, along with blood biochemistry and molecular markers detection. Thus, microfluidic technologies can be used to obtain a variety of interesting applications, such as PCR amplification and electrophoresis [38,39], immunoassays and flow cytometry [40,41], proteins for analysis via mass spectrometry [42], DNA analysis [43], chemical gradient formation [44], cell manipulation and separation [27,45,46], cell patterning [47] and single-cell analysis [25,48,49,50,51,52], NMR [53,54,55], electrochemical [56,57] and measurements such as fluid viscosity [58,59], and pH [60,61] of the blood samples and its constituents.

Separation of cells/molecules or other fluid elements plays also an important role in sample preparation for biological, biochemical, and pharmaceutical applications [24]. Additionally, cell sorting and separation need to be carried out precisely to develop microfluidic systems as an accurate tool with high detection and quantification efficiency, leading to an efficient single-cell analysis [62].

Different kinds of single-cell analysis can be performed depending on what we are observing, e.g., single-cell immunology, single-cell biology, single-cell systems biology, single-cell pharmacology, and single-cell toxicology. For instance, single-cell studies require cells capture/isolation using different microfluidic methods, such as hydrodynamic, electrical, optical, magnetic, and acoustic methods [62], and various detection methods, such as fluorescence microscopy, fluorometry and mass spectroscopy, optical or electrochemical [62] that can be combined with passive [27,28,63], active [29,64,65] or/and hybrid isolation methods [66]. As for either single-cell manipulation or single-cell analysis, it is hard to obtain a comprehensive result by merely using one method [62]. Therefore, two or more methods are usually combined into a microfluidic system comprising several single-cell testing modules [22,67].

RBCs’ deformability (the ability to change their shape and pass through small capillaries and splenic sinuses), a single-cell measurement, has been considered a potential biomarker for blood disorders, such as diabetes [68], obesity [69], and malaria [70,71,72]. Depending on the diseases, several alterations can occur in the viscoelasticity of the membrane of the cell or in the cytoplasmic viscosity, or even both. For example, nonenzymatic glycation of several proteins, especially red cell-membrane glycoproteins and hemoglobin, has been found in patients with diabetes, and such a biochemical modification of the erythrocyte is one factor that may account for altered rheological properties of human erythrocytes in diabetes [19]. Reduction in cancer-cell elasticity and stiffness-sensing ability could cause the loss of cancer cells to response to microenvironmental changes and it was suggested as important biomarkers of a cancer-cell phenotype, mechanosensation, or mechanotransduction [17]. Other diseases that include changes in RBCs stiffness due to cytoskeletal modifications such as spherocytosis, increased cell deformability of invasive cancer cells compared to benign or normal cells of the same origin, and changes in stiffness of leukocytes in response to activation with antigens or other signals. An increased deformability has also been identified as a potential biomarker for pluripotent stem cells.

Primarily, the available methods to measure the biomechanical properties of RBCs, such as the conventional rotational viscometer, ektacytometer, and micro-pore filtration assay, use high sample concentrations, since they have been used to measure the blood viscosity and other rheological properties, but they are generally expensive, labor-intensive, and do not provide a direct and detailed source of information on the mechanical properties of the RBCs. As single-cell techniques, micropipette aspiration, and optical tweezers, are also extremely popular for measuring the mechanical properties of the RBC membrane.

However, these techniques also have several drawbacks, such as a low-throughput, labor-intensive, and static process. Additionally, it is argued that these methods do not represent the actual RBC deformability that happens during microcirculation [5,50]. For this reason, the deformability of RBCs, by using microfluidic devices to measure the deformation of blood cells, has gained great attention.

### RBCs Deformability in Microfluidic Devices

**Figure 2:**
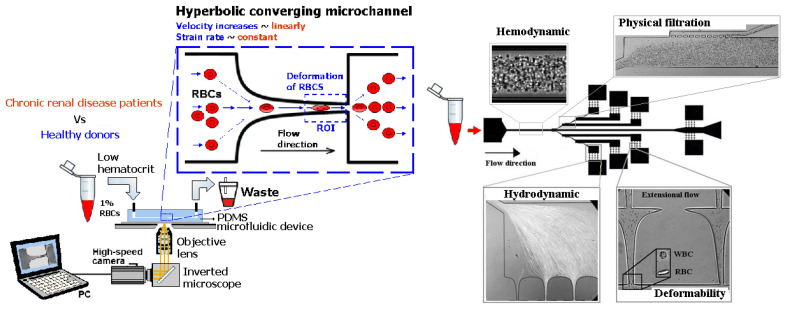
Microfluidic devices to assess of motions and deformations of red blood cells (RBCs) from healthy donors and pathological patients, e.g., chronic renal disease and diabetes mellitus [50]. ROI—Region of interest; PDMS—Polydimethylsiloxane; WBCs—White blood cells; RBCs—Red blood cells.

As previously mentioned, malaria and diabetes are some of the several existent RBCs-related diseases that can promote significant alterations in the RBC deformability. For this reason, the deformability of RBCs has been extensively studied and, recently, microfluidic devices have become the preferred method to measure the deformation of blood cells due to their dimensional match with biological cells and the aforementioned advantages. A summary of the main features of several cell deformability studies performed in microfluidic devices is presented in Table 1. Lima’s and Lee’s research group have applied a hyperbolic shaped microchannel [50,51,73,74] to study the RBCs deformability demonstrating that single RBCs deformability analysis can provide more precise and detailed information about blood disorders. In a more recent study performed by Faustino et al., 2018 [23], based in the work of Pinho et al., 2013 [26] and Rodrigues et al., 2015 [27], they have combined several passive sorting methods. In this work, separation by the biomechanical cells properties, hydrodynamic phenomena and hemodynamic cells behavior were fully integrated in a single microfluidic system to achieve single RBC visualizations and respective mechanical properties of cells (i.e., deformability) analysis. In addition, the separation efficiency was quantified by a spectrophotometric method. Briefly, this integrated microfluidic platform comprises several stages of cross-flow filtration barriers with different gaps and nine different outlets in order to access partial separation of RBCs and consequentially their deformability. At each outlet, a sequence of hyperbolic channels for cells visualization and deformability assessments is presented, which simultaneously can assess RBCs, WBCs, and other kinds of cells. The cells’ deformability was obtained, due to the use of hyperbolic-shaped contractions, which generate constant strain-rates that makes this tool a promising strategy for measuring RBCs deformability under a well-controlled microenvironment. More detailed information about this device can be found at the work performed by Faustino et al., 2018 [23]. Another example, showing the potential of this microfluidic approach, was the work performed by Rodrigues et al., 2016 [73]. In this study, this methodology was used to evaluate the impact of nanoparticles on human RBCs. The applied microfluidic tool has shown that a small amount of nanoparticles can affect the RBC deformability, where other hemocompatibility tests (such as the hemolysis analysis) did not show any influence. Hence, this study has shown that this microfluidic tool has a higher sensitivity to detect small changes of RBC deformability, task not possible by using conventional hemocompatibility methods. This microfluidic tool is currently being applied in novel studies including pathological flow studies and hemocompatibility tests (Figure 2).

In addition to the previous investigations, an innovative micro-device to study the cell deformability has been developed by Rubio et al., 2019 [75]. In this study, the authors developed fire shaped axisymmetric borosilicate micro nozzles whose production is fast, simple, and use low-cost equipment, comparing with other similar microfluidic devices found in the literature. Moreover, the shape of the converging area of the micro nozzles allowed to obtain an extensional flow, which is proper to produce a controlled deformation or elongation of microparticles/cells to measure the deformation index (DI). Although this is a preliminary study, the use of these types of micro nozzles have great potential to detect small changes of blood cell mechanical properties and it may become an interesting platform to investigate new treatments associated with pathological alterations of blood cell properties.

**Figure 3:**
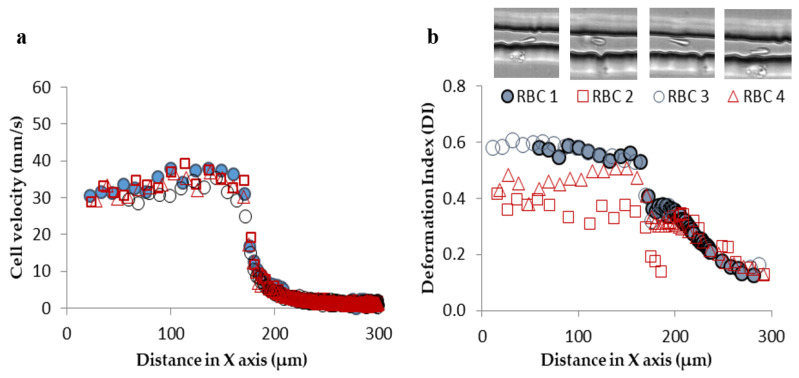
Individual RBCs (**a**) velocity flowing through a sudden contraction microchannel for a flow rate of about 1 µL/min and (**b**) deformation index (DI).

Despite the variety of experimental microfluidic studies performed to evaluate the RBCs’ deformability, some considerations must be taken into account during the flow measurements. For instance, in a study conducted in a hyperbolic converging microchannel, the effect of orientation of cells on the velocity and deformation was analyzed. As can be observed in Figure 3a, the velocity is almost the same for all RBCs, however, the deformation of the cell is dependent on its orientation and shape (Figure 3b). Depending on the orientation and shape, blood cells will have different deformability indexes. Therefore, attention must be taken in choosing the cell to assess the deformability. In this way, automatic methods may not be the most appropriate approach to measure individual blood cells, as they do not have this sensibility yet and, therefore, the results may not be as reliable as the manual methods.

Most of the works have been developed around the analysis of blood-based biomarkers. However, precise information about malignancies can also be found through liquid biopsies [76] by isolating and analyzing rare cells, classical protein biomarkers, exosomes, circulating-free nucleic acids, tumor-derived vesicles or proteins, and metabolites [77].

Despite all this physical and hydrodynamic methods, due to the simplicity of electrical measurement, measuring RBC deformability electrically was also attempted in microfluidic systems. Zeng et al., 2013 [78] measured electrically the deformability of RBCs with a two-stage microdevice. A low-frequency voltage signal was established across the microfluidic channel, and electrical current signal was sampled continuously when RBCs passed through a small microchannel [78].

**Table 1:** Main features of several cell deformability studies performed in microfluidic devices.

Microfluidic Device	Cell Types	Main Flow Phenomenon	Approach to Measure the Degree of Deformability	Configuration	Main Key Observations	References
Hyperbolic converging microchannels	Human RBCs (healthy and diseased)	Extensional flow	Deformation Ratio (DR)=$\frac{L}{D}$ 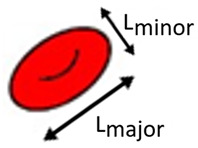	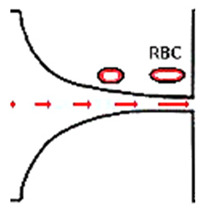	The proposed device is able to detect changes in DR of the RBCs, allowing for distinguishing the samples from the healthy controls and the patients.	Faustino et al., 2019 [50]
Hyperbolic converging microchannels	Human RBCs with magnetic NPs	Extensional flow	Deformation Index (DI)=$\frac{X-Y}{X+Y}$ 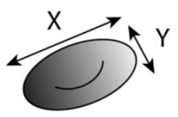	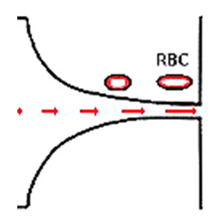	This microfluidic tool is capable of evaluating with high accuracy the impact of multifunctional nanoparticles designed for theranostic applications in contact with RBCs, using the proved extensional flow approach to measure with high accuracy the RBC’s DIs.	Rodrigues et al., 2016 [73]
Cross-flow microfluidic device with pillars	Human RBCs (healthy)	Cross-flow	Deformation Index (DI)=$\frac{X-Y}{X+Y}$ 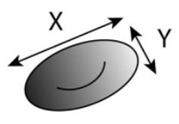	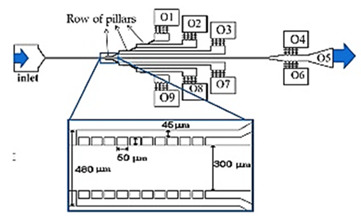	The proposed microfluidic device has the potential to perform in one single step a partial passive separation of RBCs based on their deformability by measuring the optical absorption of the collected samples.	Faustino et al., 2018 [23]
Hyperbolic converging microchannels	Human RBCs (healthy and and artificially impaired)	Shear and extensional flow	Deformation Index (DI)=$\frac{X-Y}{X+Y}$ 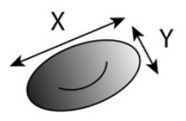	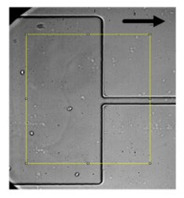	This work is a valuable contribution to help establishing the development of new malaria diagnostic systems towards point-of-care devices.	Vilas Boas et al., 2018 [74]
Fire-shaped cylindrical glass micronozzles	Human RBCs (healthy and and chemically treated)	Extensional flow	Deformation Index (DI)=$\frac{X-Y}{X+Y}$ 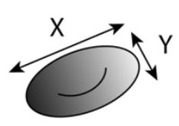	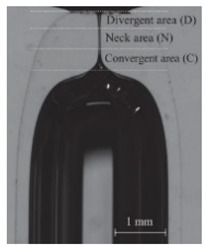	The use of these types of micronozzles, whose fabrication is simple, fast, and uses low-cost equipment, to assess the deformability (DI) of microentities, have great potential to detect small changes of blood cell mechanical properties.	Rubio et al., 2019 [75]
Polydimethylsiloxane (PDMS) rectangular abrupt contractions	Human RBCs (healthy and chemically treated)	Shear flow	Deformation Index frequency of 10 kHz	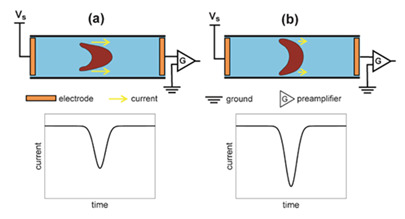	The RBC with higher deformability is more stretched along the flow direction (a), resulting in larger gaps between the RBC membrane and channel walls than the less deformable RBC (b). RBC with higher deformability blocks less current than RBC with lower deformability.	Zeng et al., 2013 [78]

## 3. Blood Analogues—Particulate Approaches

Human blood is a multiphase complex fluid that consists of a concentrated suspension of formed cellular elements (RBCs, WBCs, and platelets) in plasma [79]. However, the manipulation of real whole blood in vitro is difficult, not only because of ethical, economical, and safety problems but also because the rheological properties of blood vary with temperature, and it is difficult to control [80,81]. For these reasons, the development of reliable blood analogues to represent and reproduce the properties of human blood, is of great importance.

In the literature, there are several proposed blood analogue fluids; however, they are mostly Newtonian fluids using mixtures of water/glycerol [82] and water/dimethylsulfoxide(DMSO) [83], or non-Newtonian analogue fluids based on aqueous solutions of xanthan gum (XG) and polyacrylamide (PAA) [84], in which the addition of glycerin is used to obtain the blood rheology at different hematocrit levels [84,85]. Early studies using particulate blood analogues were developed by Fukada et al., 1989 [86], who studied the rheological properties of polystyrene microsphere suspensions with Newtonian behavior and, by adding dextran and calcium chloride into the suspensions, a non-Newtonian behavior was obtained similar to that observed for human blood. Another early work was performed by Liepsch et al., 1991 [87], in which four non-Newtonian fluids were studied in order to determine how closely they simulate the flow behavior of human blood. The viscous and viscoelastic properties of these fluids were compared with human blood samples in steady and transient Couette flow and in an oscillatory tube flow. In the end, they verified that all fluids closely mimics the flow behavior of blood. Lerche et al., 1993 [88] studied different PAA solutions with different molecular weights and also with different concentrations, and they observed that all solutions are non-Newtonian and exhibit shear-thinning behavior. They stated that PAA solutions of appropriate concentration and molecular weight should be used to mimic the non-Newtonian steady shear viscosity of normal blood. As a continuation of the previous work, Vlastos et al., 1997 [89] compared the rheology of PAA and XG solutions at different concentrations for the preparation of blood analogue solutions and performed a combination of steady and oscillatory shear tests and found that the PAA (AP 273E) solution displayed a viscoelastic behavior close to that of blood at a concentration of 125 ppm and the XG at 500 ppm, at low shear regions, At the transition and higher shear regions, all model fluids studied at the above concentrations presented higher viscous and elastic components than blood.

Sousa et al., 2011 [84] compared the flow of a Newtonian fluid and two well-established non-Newtonian blood analog polymer solutions of PAA and XG. They observed that despite their similar shear rheology, the flow patterns of the two blood analog solutions are considerably different and verified that the polyacrylamide solution exhibited a much stronger elastic character, demonstrating that elastic properties of the fluid have a major impact on the flow characteristics, so they need to be considered in the development of reliable blood analogues. Campo-Deaño et al., 2013 [81] developed four different polymer solutions (PAA+hyaluronic acid (HA)+DMSO; XG+DMSO; PAA+HA+sucrose; XG+sucrose) with different refractive indices (1.39 and 1.41) as viscoelastic blood analogues and they observed that the rheological behavior of the analogues obtained from steady shear and passive microrheology experiments is in good agreement with the rheological properties of whole human blood. Walker et al., 2014 [90] investigated the flow of a non-Newtonian blood analog of an aqueous solution of XG, on flow separation in both steady and pulsatile flow, and verified that the XG solution enhances the flow stabilization, which in turn emphasizes the importance of non-Newtonian blood characteristics on the resulting flow patterns in the presence of stenosis.

**Figure 4:**
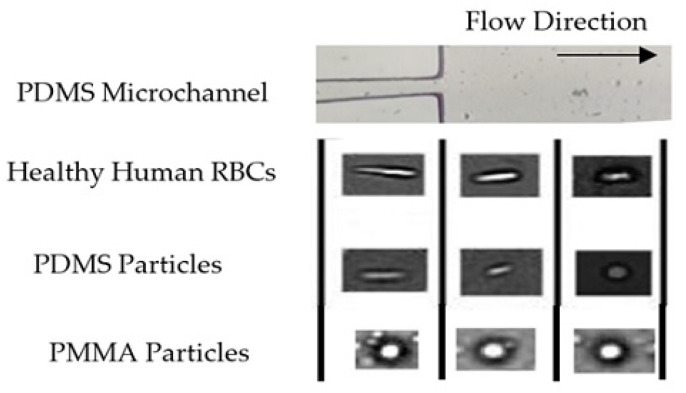
Behavior of healthy RBCs, flexible particles (PDMS) and rigid particles (PMMA) when flowing through a constriction in a microchannel. Adapted from [91,95].

**Figure 5:**
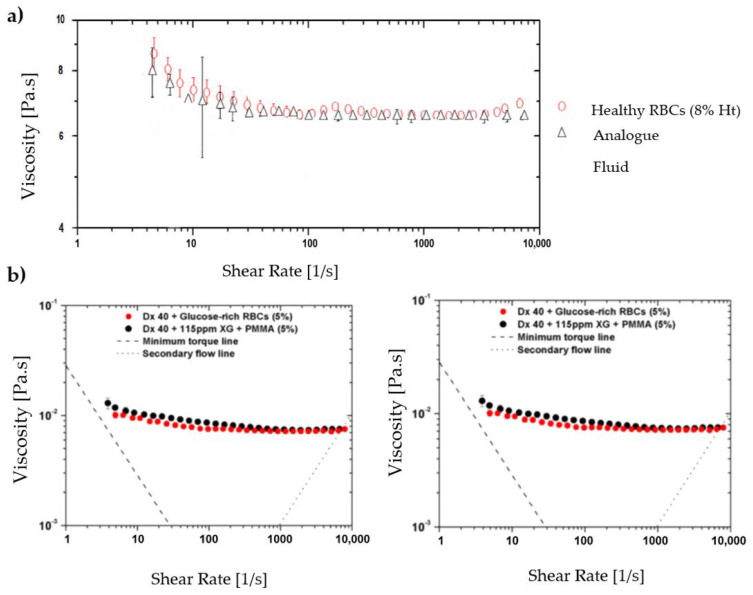
Comparison between the viscosity of a blood solution and the analogue fluid with (**a**) PDMS particles and (**b**) xanthan gum and PMMA particles. Adapted from [79,95].

Calejo et al., 2016 [91] and Pinho et al., 2017 [92] performed a study in which particulate Newtonian and non-Newtonian blood analogues were successfully developed, able to form the cell-free layer (CFL) downstream of a microfluidic hyperbolic contraction and to mimic the viscosity behavior of RBCs. Both solutions were composed of dextran 40 with rigid spheres particles of PMMA, and one of them exhibited a viscoelastic behavior due to the addition of XG. Nevertheless, the use of rigid particles limits the physiological realism of these studies. To overcome this limitation, over the years, several researchers have investigated other alternatives such as, polydimethylsiloxane (PDMS) microparticles [93,94,95] and hydrogel microparticles [96]. An example of the suitability of PDMS microparticle was presented by Pinho et al., 2019 [95]. They developed flexible PDMS microparticles to mimic RBCs in blood particulate analogue fluids and these showed a great potential to mimic the structural and rheological properties of RBCs suspensions and consequently to develop blood analogue fluids similar to real blood. The authors visualized the flow through a hyperbolic-shaped contraction and observed that all PDMS microparticles present some ability to elongate when passing through the smallest dimension of the microchannel. Figure 4 shows the deformation behavior of PDMS particles in comparison to RBCs and rigid particles of PMMA. They also verified that the final working fluid, formed of transparent PDMS microparticles, can reproduce the viscosity curve of human RBCs suspensions, showing an intermediate shear-thinning degree between healthy and pathological RBCs. The comparison between the viscosity of the working fluid and human RBCs suspensions is presented in Figure 5.

Regarding the hydrogel microparticles, Chen et al., 2012 [97] synthetized deformable microgel particles loaded with bovine hemoglobin (Hb) mimicking the RBCs’ shape and size by using the PRINT (particle replication in nonwetting templates) technique. These particles demonstrated excellent deformability to pass through restricted pores half as wide as the diameter of the particles, and showed similar viscosity to that of mouse blood.

In addition to the previous studies, another innovative blood analogue containing giant unilamellar vesicles (GUVs) with dimensions and mechanical properties similar to real RBCs was proposed by Carvalho et al., 2018 [98]. However, this method still needs improvements related to both visualization and the number of vesicles produced. Regardless the fact that all of these different blood analogues, summarized in Table 2, were able to reproduce the rheological properties of blood, only few are focused on the ability to reproduce the hemodynamic phenomena occurring in the blood flow at microcirculation scale under healthy or pathological blood conditions. It is important to achieve a high comprehension of healthy and pathological blood rheology, in particular the influence of RBCs lower deformability in the blood viscosity. In addition, the development of blood analogue fluids capable of mimic the RBCs behavior and the blood microcirculation phenomena at in vitro conditions is crucial. These analogue fluids are extremely important for experimental work since they minimize the use of real blood and the ethical issues. Lima and his colleagues have proposed a blood analogue fluid composed of Brij L4 [99] surfactant micelles suspended in pure water. This analogue is extremely easy to be produced and is able to investigate different kinds of microscale blood flow phenomena in simple and complex geometries. In addition, by using this analogue it is possible to track the flexible micelles avoiding sedimentation, aggregation, clogging and blockage difficulties that researchers have been pointed out by using rigid microparticles.

In Table 2, it is clear that the analogue fluids do not present all the rheological and mechanical properties of the real whole blood at both micro and macro scale level. However, considering the desired application, most of the developed fluids have achieved their main purpose, for example in the case of the particulate ones, the flexible microparticles with diameters similar to RBCs, are able to mimic their deformability behavior when passing through narrow microfluidics contractions, at both pathological and healthy conditions.

## 4. Future Perspectives

The main idea of personalized medicine is the concept of a patient-centered care and in this review, we have shown and discussed several tools that can couple with several detection methods and applied to specific patient needs in order to individually assess each problem. Thus, even for something more personalized or for a more general screening tools, microfluidic technology progress has promoted the ability to not only precisely control and manipulate fluids but it also has allowed medical researchers to develop clinical platforms for rapid sample analysis. The unique features of microfluidics are valuable for the progress of individualized medicine plans such as new treatment protocols and diagnosis approaches. Recently, individualized medicine research has been explored for applications such as point-of-care testing and individualized drug therapy. By applying microfluidic technologies, it is expected the improvement of the effectiveness in detecting biomolecules and monitoring individualized diagnosis and therapy studies in vitro and in vivo. The visualization and measurements of blood cells, flowing in microfluidic devices have been proven to be important in providing not only essential information about hydrodynamic characteristics of the blood, but also vital information to diagnose early symptoms of diseases during clinical investigations. For instance, point-of-care devices combined with flow visualization techniques, can be used to assess the cardiac risk profile of a specific patient and help the medical staff to decide the appropriate treatment and consequently to increase the chances of survival of this patient. Moreover, the new generation of blood analogue fluids, with the ability of reproducing healthy or pathological conditions, have been demonstrated to be a promising approach to improve the knowledge about several blood diseases. Ideally, particulate solutions having flexible particles that mimic key structural attributes of RBCs including size, shape, and mechanical properties would be an excellent candidate to reproduce multiphase effects of the blood flow in microcirculation.

From a clinician’s perspective, it is still unclear about the translational part in particular with regard to personalize medicine. However, there are clear evidences that single cells analysis, in particular of RBCs provide a unique and specific information about the patient stratification, for example the overall outcome of clinical diabetes care and management.

## Figures and Tables

**Table 2:** Main fluids and particles used to mimic blood and RBCs behavior.

Analogue Fluid	Main Key Observations in Comparison to Whole Blood	References
Glycerol and water	Newtonian, macro behavior, viscosity curve	Lieber et al., 2009 [82]
Water and DMSO	Newtonian, macro behavior, viscosity curve	Carvalho et al., 2020 [83,100], Souza et al., 2020 [101]
Xanthan gum (XG) and PAA XG, PAA and glycerin	Non-Newtonian, macro behavior, hematocrit level, shear-thinning	Sousa et al., 2011 [84]
Dextran 40 and CaCl_2_	Non-Newtonian, cell-free layer, micro behavior, hemodynamic phenomena, shear-thinning	Fukada et al., 1989 [86]
PAA	Non-Newtonian, shear-thinning behavior	Lerche et al., 1993 [88]
PAA and XG	Non-Newtonian, shear-thinning behavior, viscoelasticity	Vlastos et al., 1997 [89]
Sucrose, PAA and HA	Non-Newtonian, macro behavior, shear-thinning behavior, viscoelasticity	Campo-Deaño et al., 2013 [81]
Sucrose and xanthan gum	Non-Newtonian, macro behavior, shear-thinning behavior, viscoelasticity	Campo-Deaño et al., 2013 [81]
PMMA	Newtonian, micro behavior, pathological RBCs deformability	Calejo et al., 2016 [91]
PMMA particles, xanthan gum, Dx 40	Non-Newtonian, micro behavior, shear-thinning behavior, viscoelasticity, pathological RBCs deformability, cell-free layer	Pinho et al., 2017 [92]
PDMS beads in Dx 40	Non-Newtonian, micro behavior, shear-tinning behavior, RBCs deformability	Pinho et al., 2019 [95]
Hydrogel	Non-Newtonian, viscoelasticity	Nguyen et al., 2004 [96]
Microgel particles loaded with bovine Hb	Newtonian, micro behavior, RBCs deformability	Chen et al., 2012 [97]
Giant unilamellar vesicles, water	Newtonian, micro behavior, RBCs deformability	Carvalho et al., 2018 [98]
Brij L4 surfactant, water	Non-Newtonian, micro behavior, two-phase, shear-thinning behavior, cell-free layer, RBCs deformability	Lima et al., 2020 [99]
